# Expression and Clinical Significance of HKII and HIF-1α in Grade Groups of Prostate Cancer

**DOI:** 10.3389/fgene.2021.680928

**Published:** 2021-06-17

**Authors:** Xueqi Sun, Qirui Huang, Fang Peng, Jian Wang, Weidong Zhao, Guangxiu Guo

**Affiliations:** ^1^Department of Pathology, Ganzhou People’s Hospital, Ganzhou, China; ^2^School of Medical Information Engineering, Gannan Medical University, Ganzhou, China; ^3^College of Computer Science and Software, Shenzhen University, Shenzhen, China

**Keywords:** prostate cancer, the Warburg-like effect, HKII, HIF-1α, grade group

## Abstract

Prostate cancer (PCA) is the second leading cause of cancer-related mortality in men. The glycolytic enzymes hexokinase II (HKII) and the major regulator hypoxia-inducible factor-1α (HIF-1α) are PCA-specific biomarkers. Some studies have shown that HKII and HIF-1α are highly expressive in PCA and are associated with the growth and metastasis of treatment. Whether HKII and HIF-1α regulate the different differentiation of PCA remains largely unknown. Therefore, the study aims to explore the value of HKII and HIF-1α in different grade groups of PCA. Our data indicated that compared with normal prostate tissues, the level of mRNA and protein of HKII and HIF-1α in PCA increased significantly, besides the results showed the high expression of HKII and HIF-1α had a tendency to promote the progression and differentiation of PCA. The study also found that HKII expression was positively correlated with the expression of HIF-1α. HKII and HIF-1α were related to the degree of differentiation PCA, especially in high-grade PCA. Furthermore, the high expression of HKII was significantly associated with Gleason score and histological differentiation in clinicopathological characteristics of patients with PCA. These results were further used to confirm that the expression of HKII and HIF-1α was associated with the progression and differentiation of PCA. These experiments indicated that HKII and HIF-1α might be novel biomarkers of PCA with potential clinical application value, provide a new potential target for PCA treatment, and are expected to be used for individualized treatment in patients with PCA.

## Introduction

Prostate cancer (PCA) is another leading cause of cancer-related mortality in men (Siegel et al., 2019). At present, the incidence and mortality of PCA in China are increasing rapidly year by year (Liu et al., 2019). Radical prostatectomy and radiotherapy are significant treatment strategies for PCA. However, the recurrence and metastasis of high-risk PCA patients limited the efficacy of radiotherapy (Du et al., 2020). Lots of studies have been conducted on the underlying pathogenesis of PCA and its related genes and proteins, aiming to improve the diagnosis and treatment of PCA metabolic rearrangement. Metabolic reprogramming is one of the hallmarks of cancer. Glycolysis is a critical catabolic process of glucose. Under normal physiological conditions, extracellular glucose would be transported cytoplasm with glucose transporter and then degraded into pyruvates catalyzed by glycolysis enzymes. Pyruvate is transformed into lactate by the lack of oxygen via an anaerobic glycolysis pathway. In contrast, pyruvate is oxidized to CO_2_ and H_2_O through the oxidative phosphorylation (OXPHOS) pathway when oxygen is sufficient, which produces amount number of adenosine triphosphate (ATP). Most tumor cells are more dependent on glycolysis to generate large amounts of lactic acid, even if there is sufficient oxygen (Yu et al., 2017), this type of aerobic glycolysis is called the Warburg effect (Warburg, 1956). The Warburg effect is a hallmark of tumor survival and growth. A large number of studies have been carried out to determine the role of the Warburg effect in the occurrence of solid tumors and its correlation with poor prognosis. However, there are few studies about the Warburg effect in patients with PCA.

Hypoxia-inducible factor-1α (HIF-1α) is a master regulator of glycolysis and plays an important role as an activator of aerobic glycolysis and lactate production. HIF-1α subunit is sensitive to oxygen concentration and has an extremely short half-life. The increase of reactive oxygen species inhibited prolyl-hydroxylases (PHD) activity and HIF-1α state was stable under hypoxia condition. Activation of HIF-1α can enhance glycolysis and decrease the tricarboxylic acid (TCA) cycle and OXPHOS, and it may stimulate tumor cell proliferation, apoptosis resistance, migration, and invasion in different tumor cell lines (Fan et al., 2016). HIF-1α mediated upregulation of hexokinase II (HKII) leads to a high rate of glycolysis in solid tumors under hypoxia (Rempel et al., 1996). Studies have demonstrated that HIF-1α is activated in diffuse large B-cell lymphoma (DLBCL) cells under hypoxia conditions, thereby inducing the expression of HKII (Bhalla et al., 2018).

HK is the first key enzyme in the control of cellular glycolysis flux. HK contains four subtypes, which HKII is the most important subtype of glucose metabolism in tumor cells. Overexpression of HKII not only increases the rate of glycolysis, but is also necessary for the occurrence of tumors. HKII is a serine protease with 79% of the same amino acid sequence as the prostate-specific antigen (PSA) and is also a novel PCA biomarker (Timmermand et al., 2014). HKII is mainly produced in the prostate gland, secret in the body in the form of proenzyme, and activated as an active enzyme outside the cell. HKII was up-regulated in malignant tumors (Mao et al., 2018).

Gleason grading system is currently the most widely used grading system for histological evaluation of PCA. Gleason grade group plays a major role in determining the prognosis of PCA. The 2014 ISUP Expert Consensus Meeting further revised the Gleason grading system for PCA (Epstein et al., 2016), this grading system not only defines the morphological criteria of PCA in Gleason more details and clearly, but also puts forward a set of new grade groups based on prognosis, which is called the grade groups system of PCA (Kryvenko and Epstein, 2016). Gleason score grouping system was endorsed which consisted of five grades, and the grade groups appeared to provide better risk stratification than the standard Gleason scoring (Spratt et al., 2016). Gleason score 6 (3 + 3) was classified as grade group 1 which better reflected the mostly indolent behavior of tumors, Gleason score 3 + 4 = 7 being ISUP grade group 2 and 4 + 3 = 7 ISUP grade group 3, Gleason scores 4 + 4 = 8, 3 + 5 = 8 or 5 + 3 = 8 were classified as grade group 4, Gleason scores 9–10 had been labeled as grade group 5. Low-grade PCA consisted of Grade groups 1 and 2, whilst high-grade PCA included grade groups 3, 4, and 5. The application of ISUP classification in prediction with the aid of some scholars has been confirmed in some studies (Humphrey, 2017).

Although studies have investigated the Warburg effect in tumors, including PCA, it is difficult to determine whether there is a definite link between the Warburg effect and PCA grade groups. The mechanism by which PCA and glycolysis induce HIF-1α and HKII is still unclear. In this study, we evaluated the role of HKII and HIF-1α for predicting PCA progression. These results will help to evaluate the role of these markers in the progression of PCA and are expected to be utilized in the individualized treatment of PCA patients.

## Materials and Methods

### Tissues Samples

A total of 129 (average: 70 ± 7 years) formalin-fixed, paraffin-embedded tissue samples were collected from the People’s Hospital of Ganzhou between October 2018 to December 2020 in this study. All specimens were available from the surgery. None had received pre-operative radiotherapy, chemotherapy, or hormone drug therapy. Patients were evaluated per 2014 ISUP Gleason grade groups in PCA. The number of cases classified as Gleason grade groups 1, 2, 3, 4, and 5 were 15, 15, 25, 30, and 24, respectively. In addition, 20 normal prostate tissues were invoked as controls. The research involving human tissues was subject to approval by the Ethics Committee of the People’s Hospital of Ganzhou, and all patients and controls were informed and consented to their participation in the study.

### Immunohistochemistry

Sections (4 μm) were obtained from formalin-fixed, paraffin-embedded tissues. Immunohistochemistry (IHC) staining was conducted with antibodies against Hexokinase II (Abcam, rabbit polygonal antibody, diluted at 1/600), Hypoxia-Inducible Factor-1 alpha (Genetex, mouse monoclonal antibody, diluted at 1/50), and horseradish peroxidase-labeled secondary antibody (Maixin Biotechnology, Fuzhou, China) in accordance with manufacturer’s instructions. The color was established with diaminobenzidine (Dako) incubated for 5–10 min at room temperature. Slides were countersigned with hematoxylin and examined by light microscopy.

Staining intensity was graded according to the following criteria described 0 (no staining), 1 (weak staining, light yellow), 2 (moderate staining, yellow with brown), and 3 (strong staining, Brown). Percent staining was graded according to the proportion of positively stained cells as follows: 0 for ≤5% positive cells; 1 for 6–25% positive cells; 2 for 26–50% positive cells and 3 for ≥ 51% positive cells. The immunoreactive score (IRS) was used to measure results. IRS = staining intensity × percent of positive cells. IRS score of 4 and higher was regarded as high expression. Ten high-power fields were randomly selected during observation, and the results were averaged. The staining results were combined with the judgment of two pathologists with rich clinical experience.

### Quantitative Real-Time Reverse Transcription PCR

Total RNA were prepared from paraffin-embedded tissues using Trizol reagent (Invitrogen Life Technology) according to the manufacturer’s protocol, reverse transcription was performed using one step PrimeScript cDNA Synthesis Kit, and quantitative real-time PCR was performed using SYBR Premix Ex Taq II (Takara Biotechnology). The primer sequences were 5′-ATTGTGGCTGTGGTGAATGA-3′ (forward) and 5′-CGCATCTCTTCCATGTAGCA-3′ (reverse) for HKII, 5′-TGCAACATGGAAGGTATTGC-3′ (forward) and 5′-TTCACAAATCAGCACCAAGC-3′ (reverse) for HIF-1α, 5′-CTTAGTTGCGTTACACCCTTTCTTG-3′ (forward) and 5′-CTGTCACCTTCACCGTTCCAGTTT-3′ (reverse) for β-actin. The relative quantity of the target mRNA was normalized to the level of β-actin mRNA level. All experiments were performed in triplicate. Expression fold-change of genes was evaluated using 2^–△△^^Ct^ (relative quantitative method), and *t*-test and one-way analysis of variance were performed.

### Statistical Analysis

IBM SPSS 22.0 and GraphPad Prism 8.0.2 were used for statistical analysis of the data. Differences of HKII and HIF-1α expression among multiple groups were analyzed by the chi-square test or Fisher’s exact test. Multiple comparisons between each two groups were examined by the Bonferroni test, the correlation was analyzed by Pearson correlation, and a *p* ≤ 0.05 was considered statistically significant. The correlation of HKII and HIF-1α expression was analyzed by the Spearman correlation test. Associations between clinicopathological characteristics and HKII or HIF-1α were observed by the chi-squared test.

## Results

### Expression of Glycolysis Enzymes in Normal Prostate Tissues and Different Grade Groups of PCA

**FIGURE 1:**
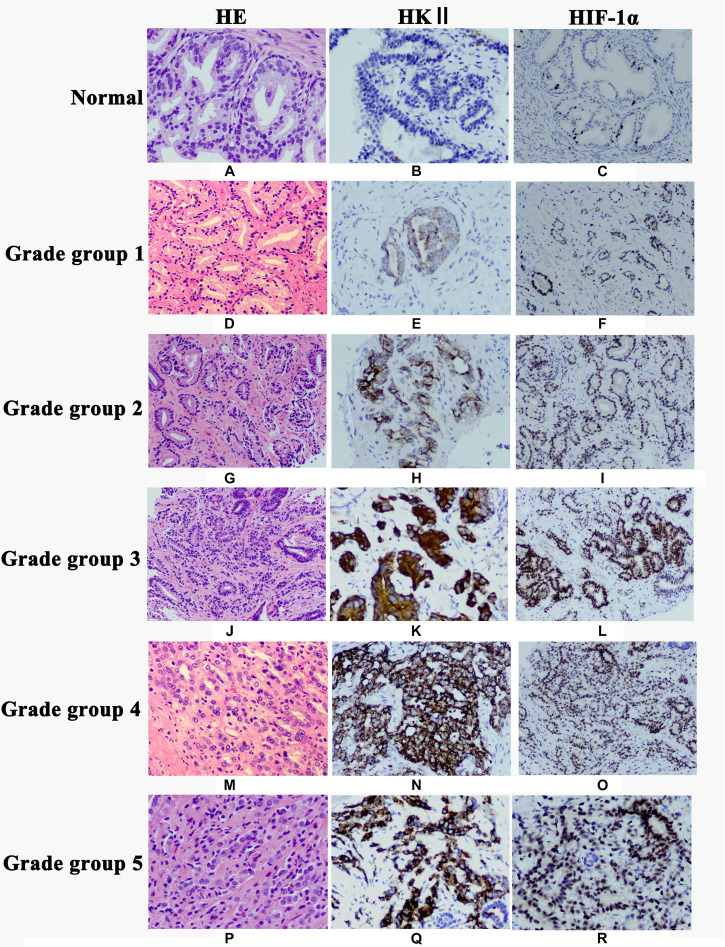
Expression of HKII and HIF-1α in normal prostate tissues, prostate cancer grade group 1, grade group 2, grade group 3, grade group 4 and grade group 5 detected by immunohistochemistry. Hematoxylin-eosin (HE) staining showed morphology of normal prostate tissues **(A)**, Grade group 1 **(D)**, Grade group 2 **(G)**, Grade group 3 **(J)**, Grade group 4 **(M)** and Grade group 5 **(P)**. HKII staining was undetectable in normal prostate tissues **(B)**. Immunoreactivity of HKII gradually increased from Grade group 1 **(E)**, Grade group 2 **(H)**, Grade group 3 **(K)**, Grade group 4 **(N)** to Grade group 5 **(Q)**. The expression of HIF-1α gradually elevated from normal prostate tissues **(C)**, Grade group 1 **(F)**, Grade group 2 **(I)**, Grade group 3 **(L)**, Grade group 4 **(O)** to Grade group 5 **(R)**.

The clinicopathologic characteristics of PCA were presented in Table 1. HKII is the key rate-limiting enzyme of glycolysis. We first profiled its expression in normal prostate tissue and different grade groups of PCA tissues by IHC. HKII was stained in the cytoplasm of epithelial cells (Figure 1). In term of immunostaining intensity, the high expression of HKII was detected in 40% grade group 1, 60% grade group 2, 68% grade group 3, 73.3% grade group 4 and 75% grade group 5 of PCA, respectively, while no strong staining was observed in normal prostate tissues (15%) (Table 2). We found that HKII high expression positive rates, gradually increased grading along with the progression of PCA. The expression of HKII was associated with PCA high Gleason score and poor tumor differentiation. The differences of high expression levels for HKII among normal controls and grade groups 3, 4, 5 in PCA were statistically significant (*P* < 0.05), especially in grade groups 4, 5 in PCA (*P* < 0.01). There was also a difference in HKII expression between well-differentiated (grade group 1) and poorly differentiated (grade groups 4 and 5 in PCA) (*P* < 0.01). The results demonstrated that the high expression of HKII was significantly associated with Gleason score and histological differentiation in clinicopathological characteristics of patients with PCA (Table 1, *P* < 0.05). Our results indicated that the association of HKII high expression with the biological behavior of PCA.

**TABLE 1:** Clinicopathological features of prostate cancer in relation to HKII and HIF-1α protein expression.

Parameters	Number (Total *n* = 109)	HKII			HIF-1α		
		High (*n* = 72)	Low (*n* = 37)	*P*	High (*n* = 68)	Low (*n* = 41)	*P*
**Age (years)**							
>70	57	39	18	0.585	38	19	0.334
≤70	52	33	19		30	22	
**Histological differentiation**							
High-grade	79	57	22	0.0292*	53	26	0.1
Low-grade	30	15	15		15	15	
**Gleason score**							
≥7	94	66	28	0.022*	61	33	0.176
<7	15	6	9		7	8	
**Histological stage**							
T1-T2	73	43	30	0.025*	40	33	0.020*
T3-T4	36	29	7		28	8	

High, high-expression; Low, low-expression. *P < 0.05.

**TABLE 2:** Expression of HKII in normal prostate tissues and different grade groups of prostate cancer tissues.

Tissues	HKII expression (%)	
	High	Low
Normal^a^	3/20 (15.0%)	17/20 (85.0%)
Grade group 1^b^	6/15 (40.0%)	9/15 (60.0%)
Grade group 2^c^	9/15 (60.0%)	6/15 (40.0%)
Grade group 3^d^	17/25 (68.0%)	8/25 (32.0%)
Grade group 4^e^	22/30 (73.3%)	8/30 (26.7%)
Grade group 5^f^	18/24 (75.0%)	6/24 (25.0%)
Total	75/129 (58.1%)	54/129 (41.9%)

^p–q^P: the difference in expression of HKII between the p and q groups. ^a–b^P > 0.05,^a–c^P > 0.05,^a–d^P < 0.05*,^a–e^P < 0.01**,^a–f^P < 0.01**, ^b–c^P > 0.05,^b–d^P>0.05,^b–e^P < 0.05*,^b–f^P < 0.05*,^c–d^P > 0.05,^c–e^P > 0.05, ^c–f^P > 0.05,^d–e^P > 0.05,^d–f^P > 0.05,^e–f^P > 0.05. High, high-expression; Low, low-expression.

**FIGURE 2:**
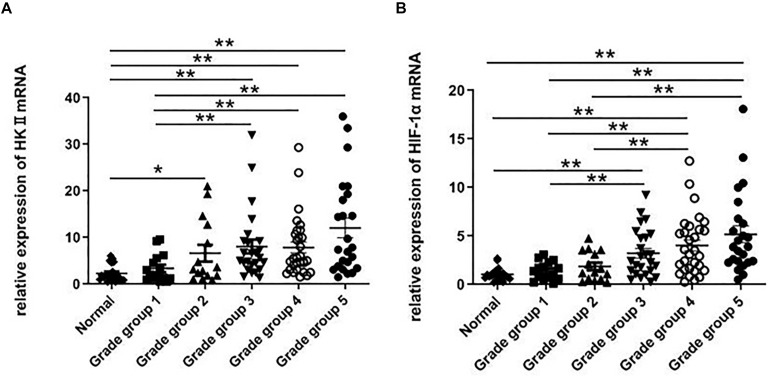
RT-PCR was used to detect the expression of HKII and HIF-1α in normal prostate tissues and different grade groups of tissues. The relative expression of HKII and HIF-1α were normalized by β-actin, the ratios of HKII/β-actin expression **(A)** and HIF-1α/β-actin expression **(B)** were calculated from triplicate (*P < 0.05, **P < 0.01).

RT-PCR was further used to confirm that during the development from normal prostate tissues to PCA Gleason grade group 1 then to PCA Gleason grade group 5, the expression of HKII mRNA also showed a trend of gradual increase. The mRNA levels of HKII in 129 prostate tissues, including 20 normal prostate tissues, 15 Gleason grade group 1 tissues, 15 Gleason grade group 2 tissues, 25 Gleason grade group 3 tissues, 30 Gleason grade group4 and 24 Gleason grade group 5 tissues were detected by RT-PCR. Levels of HKII in PCA Gleason grade group 2 were higher than that in normal prostate tissues (*P* < 0.05). This statistical difference was more pronounced in grade groups 3, 4, and 5 in PCA (*P* < 0.01). Besides, the expression of HKII mRNA in PCA Gleason grade groups 3, 4, 5 was also higher than those in grade group 1 tissues (*P* < 0.01) (Figure 2A). Ratios of HKII/β-actin expression increased along with the degree of differentiation of PCA.

### Up-Regulation of HIF-1α Expression in PCA

HIF-1α is a significant transcription factor for tumor cell energy metabolism. HIF-1α is closely related to glycolysis. The expression of HIF-1α protein was once detected through IHC. And the HIF-1α protein used to be localized in the nucleus of epithelial cells (Figure 1). The results of IHC showed the expression of HIF-1α in PCA was higher than that in normal prostate tissues (*P* < 0.05) (Table 3). Whilst the expression of HIF-1α in PCA Gleason grade group 4 was significantly up-regulated compared with that in Gleason grade group 1 (*P* < 0.05), suggesting that the expression of HIF-1α was statistically significant in PCA differentiation.

**TABLE 3:** Expression of HIF-1α in normal prostate tissues and different grade groups of prostate cancer tissues.

Tissues	HIF-1α expression (%)	
	High	Low
Normal^a^	2/20 (10.0%)	18/20 (90.0%)
Grade group 1^b^	7/15 (46.7%)	8/15 (53.3%)
Grade group 2^c^	8/15 (53.3%)	7/15 (46.7%)
Grade group 3^d^	13/25 (52.0%)	12/25 (48.0%)
Grade group 4^e^	23/30 (76.7%)	7/30 (23.3%)
Grade group 5^f^	17/24 (70.80%)	7/24 (29.2%)
Total	70/129 (54.3%)	59/129 (45.7%)

^p–q^P: the difference in expression of HIF-1α between the p and q groups. ^a–b^P < 0.05*,^a–c^P < 0.01**,^a–d^P < 0.01**,^a–e^P < 0.01**,^a–f^P < 0.01**, ^b–c^P > 0.05,^b–d^P > 0.05,^b–e^P < 0.05*,^b–f^P > 0.05,^c–d^P > 0.05,^c–e^P > 0.05, ^c–f^P > 0.05,^d–e^P > 0.05,^d–f^P > 0.05,^e–f^P > 0.05. High, high-expression; Low, low-expression.

The HIF-1α expression is associated with the progression and differentiation of PCA. When HIF-1α mRNA was examined using RT-PCR in normal prostate tissues and different grade groups of PCA, levels of HIF-1α in PCA (Gleason grade groups 3, 4, and 5) were higher than those in normal prostate tissues (*P* < 0.01). Compared with low-grade of PCA (Gleason grade groups 1 and 2), the expression of HIF-1α mRNA in high-grade of PCA (Gleason grade groups 4 and 5) increased (*P* < 0.01) (Figure 2B). The expression of HIF-1α indicated no statistically significant change in PCA Gleason grade groups 4 and 5 tissues.

### HKII Expression Was Positively Correlated to the Expression of HIF-1α

To investigate whether HKII expression is related to HIF-1α expression. We found that more than 80% (16/20) of normal prostate tissues were a low expression both for HKII and HIF-1α, both the high expression in normal prostate tissue instead of only 5% (1/20) (Table 4). Both the HKII and HIF-1α were highly expressed by up to 66.7% (20/30) in PCA tissues. Increased intensity of both HKII and HIF-1α staining were also observed along with the increased progression and differentiation of PCA. Expression of HKII in PCA Gleason grade groups 4 and 5 were positively correlated with the expression of HIF-1α (*P* < 0.05). The results indicated that the expression of HIF-1α was positively correlated with the expression of HIF-1α. Although the high expression of HKII and HIF-1α in PCA increased with the progression and differentiation of PCA, it was not statistically significant in well-differentiated PCA, which is worthy of our investigation.

**TABLE 4:** Co-relationship of HKII level with HIF-1α expression in normal prostate tissues and different grade groups of prostate cancer tissues.

HKII	Normal (*n* = 20)		Grade group 1 (*n* = 15)		Grade group 2 (*n* = 15)		Grade group 3 (*n* = 25)		Grade group 4 (*n* = 30)		Grade group 5 (*n* = 24)	
	Low	High	Low	High	Low	High	Low	High	Low	High	Low	High
**HIF-1α**												
Low	16	1	5	4	4	2	6	2	5	3	4	2
High	2	1	3	3	3	6	6	11	2	20	3	15
*P*	0.28		0.83		0.31		0.097		0.007**		0.038*	

High, high-expression; Low, low-expression. *P < 0.05, **P < 0.01.

## Discussion

In China, the incidence of PCA has been at the forefront of male malignancies. Prostate biopsy is utilized to diagnose PCA. However, there is the absence of simple and accurate indicators to monitor and guide the prognosis of PCA and individualized treatment after diagnosis. PSA serum stage and Gleason grading on histological specimens are presently the classical prognostic factor, however, they are regularly unable to predict a precise disease progression.

Warburg discovered that cancer cells are more dependent on glycolysis for survival and growth nearly a century ago, even when there is enough oxygen, now known as the Warburg Effect. The Warburg effect is considered a major marker of tumor cell metabolism and promotes the occurrence and development of tumors. The Warburg effect has long been associated with hypoxia, but it’s not entirely adapted to hypoxia, and it can also occur in non-toxic conditions (Finley et al., 2011). Persistent hypoxia can additionally enhance local and systemic malignant progression and may also extend increase invasiveness through the clonal selection and genomic and proteomic changes (Dachs and Chaplin, 1998). HIF-1α is a major regulator of glycolysis (Shen et al., 2020). Under hypoxia, HIF-1α plays an important role in the transcription of genes involved in angiogenesis, tumor growth, invasion, metastasis, and glucose metabolism (Soni and Padwad, 2017). The expression of HIF-1α in PCA cells is significantly increased, which is closely related to the proliferation and metastasis of PCA (Nenu et al., 2017; Wang et al., 2019). HIF-1α facilitate the transcription of HKII. HKII is the first rate-limiting enzyme in glycolysis. Abnormal expression of HKII is associated with the progression of a variety of tumors. B7-H3 promotes glycolysis and drug resistance of colorectal cancer cells by up-regulating the level of HKII (Shi et al., 2019). DeWaal et al. (2018) demonstrated that deficiency in HKII inhibited glycolysis metabolism of hepatocellular carcinoma and promoted OXPHOS and chemotherapy-sensitivity of hepatocellular carcinoma. A large number of experiments have confirmed that serum HKII is helpful for the detection and prognosis of PCA (Mao et al., 2018). It further shows that tumors rely on glycolysis to survive.

Commonly, most cancers cells hold an excessive glycolysis flux to meet the strength and intermediates wished for survival and fast boom. Compared with adjoining non-tumor tissues, many glycolysis enzymes are up-regulated in tumors (Lee et al., 2018). In this study, the expression of glycolysis-related enzymes HKII and major regulator HIF-1α increased in PCA. We found HKII and HIF-1α increased in PCA, further support metabolic reprogramming to the Warburg effect in tumor cells. HIF-1α regulation in PCA could provide better outcomes and treatment chances for men with PCA. Various classes of HIF-1α inhibitors have been developed and studied clinically (Semenza, 2012). The extent of tumor differentiation and the role of the Warburg effect in carcinogenesis have not been well studied.

Univariate Cox regression analysis showed that clinical stage and Gleason score were highly correlated with recurrence (Saedi et al., 1998). Furthermore, whether the elevation of HKII after prostatectomy was a better predictor of recurrence than before prostatectomy (Guerrico et al., 2017). Therefore, the search for effective biomarkers in the prediction of PCA progression survival and prognosis has an indispensable role. In our study we found that both mRNA and protein expression in HKII were significantly correlated with those in low-grade PCA and high-grade PCA, suggesting that HKII might have a dependent clinical value for tumor differentiation. HIF-1α levels were higher in high-grade PCA than in low-grade PCA at the mRNA level, however, this phenomenon could have not yet been fully demonstrated to the protein level, and only the PCA grade groups between 1 and 4 showed significant differences. IHC method may lead to different assays due to the specificity and epitope recognition of antibodies and complex formation with other molecules, and the use of other methods may help to better improve and standardize the analysis of markers for measured samples (Klee et al., 2014). These results suggest that HKII and HIF-1α could be good markers for high-risk PCA.

Cao et al. found that HIF-1α was positively correlated with the expression of HKII, bioinformatics analysis indicated that HIF-1α has a potential hypoxia response element in the upstream promoter region of HKII, HIF-1α could bind to position 1 of the HKII promoter (Cao et al., 2020). HIF-1α could target the promoter of HKII to accelerate its transcription (Feng et al., 2020). Transcription factor HIF-1α could bind with the HKII promoter, initiate HKII expression and eventually promote tumor progression. Based on these results, we hypothesized that there is an interaction between HIF1α and HKII, which were further confirmed by correlation analysis of the IHC protein level. Our data demonstrated that there was a significant correlation between HIF-1α and HKII in high-grade PCA. It is suggested that the combination of HIF1α and HKII may regulate the growth of tumor cells by promoting the glycolysis of PCA cells. HIF-1α was positively correlated with pyruvate kinase (PK) and lactate dehydrogenase A (LDHA) expression levels in aerobic glycolysis, suggesting that HIF-1α is a key transcriptional factor of aerobic glycolysis (Xia et al., 2020). Thus, increased transcription leads to high protein expression of these glycolysis components, which ultimately promote aerobic glycolysis in PCA.

The heterozygosity of tumors is excellent, and the pathogenesis of distinctive tumors is additionally diversified. Cancer development is a complicated process that is not yet fully understood. Some results obtained by RT-PCR were not completely consistent with the results obtained by IHC analysis. The sample size may have been a contributing factor. It is suggested that the key rate-limiting enzymes and key regulatory factors in glycolysis are related to the differentiation degree of PCA, especially the expression of HKII and HIF-1α in high-grade PCA. Our results furnish potential proof for the Warburg effect in the degree of tumor differentiation and provide novel potential targets for PCA treatment. Further researches are needed to evaluate or verify this phenomenon.

## Data Availability Statement

The original contributions presented in the study are included in the article/supplementary material, further inquiries can be directed to the corresponding author/s.

## Ethics Statement

The studies involving human participants were reviewed and approved by the Ethics Committee of the People’s Hospital of Ganzhou. The patients/participants provided their written informed consent to participate in this study.

## Author Contributions

XS did experiments, interpreted the data, and wrote the manuscript. QH did data analysis and provided advice and critically read the manuscript. FP and JW collected some tissues samples and provided advice. WZ provided advice and critically read the manuscript. GG designed the study, provided advice, and critically read the manuscript. All authors contributed to the article and approved the submitted version.

## Conflict of Interest

The authors declare that the research was conducted in the absence of any commercial or financial relationships that could be construed as a potential conflict of interest.
